# The Relationship Between Lycopene and Metabolic Diseases

**DOI:** 10.3390/nu16213708

**Published:** 2024-10-30

**Authors:** Anna Kulawik, Judyta Cielecka-Piontek, Bogusław Czerny, Adam Kamiński, Przemysław Zalewski

**Affiliations:** 1 Department of Pharmacognosy and Biomaterials, Faculty of Pharmacy, Poznan University of Medical Sciences, 3 Rokietnicka St., 60-806 Poznań, Poland; anna.kulawik@student.ump.edu.pl (A.K.); jpiontek@ump.edu.pl (J.C.-P.); 2 Phytopharm Klęka S.A., Klęka 1, 63-040 Nowe Miasto nad Wartą, Poland; 3 Department of Pharmacology and Phytochemistry, Institute of Natural Fibres and Medicinal Plants, Wojska Polskiego Str. 71b, 60-630 Poznań, Poland; 4 Department of General Pharmacology and Pharmacoeconomics, Pomeranian Medical University in Szczecin, 71-210 Szczecin, Poland; boguslaw.czerny@pum.edu.pl; 5 Department of Orthopedics and Traumatology, Independent Public Clinical Hospital No. 1, Pomeranian Medical University in Szczecin, Unii Lubelskiej 1, 71-252 Szczecin, Poland; adam.kaminski@pum.edu.pl

**Keywords:** lycopene, metabolic syndrome, obesity, type 2 diabetes

## Abstract

**Background:** Metabolic syndrome, obesity, and type 2 diabetes are closely related. They are characterized by chronic inflammation and oxidative stress. Obesity is the most important risk factor for metabolic syndrome and type 2 diabetes. Metabolic syndrome is characterized by insulin resistance and elevated blood glucose levels, among other conditions. These disorders contribute to the development of type 2 diabetes, which can exacerbate other metabolic problems. **Methods:** Numerous studies indicate that diet and nutrients can have a major impact on preventing and treating these conditions. One such ingredient is lycopene. It is a naturally occurring carotenoid with a unique chemical structure. It exhibits strong antioxidant and anti-inflammatory properties due to its conjugated double bonds and its ability to neutralize reactive oxygen species. Its properties make lycopene indirectly affect many cellular processes. The article presents studies in animal models and humans on the activity of this carotenoid in metabolic problems. **Results:** The findings suggest that lycopene’s antioxidant and anti-inflammatory activities make it a promising candidate for the prevention and treatment of metabolic syndrome, obesity, and type 2 diabetes. **Conclusions:** This review underscores the potential of lycopene as a beneficial dietary supplement in improving metabolic health and reducing the risk of associated chronic diseases. The conditions described are population diseases, so research into compounds with properties such as lycopene is growing in popularity.

## 1. Introduction

The development of metabolic illnesses, such as increased glucose intolerance, high blood pressure, and hyperlipidemia, is mainly caused by oxidative stress and chronic inflammatory conditions [1,2,3]. Both oxidative stress and inflammation play a significant role in the onset and progression of metabolic dysfunction. Chronic inflammation promotes metabolic disorders, making it a key target for preventing and treating these widespread diseases. Identifying nutritional strategies is a promising approach to limit the impact of metabolic diseases [4,5].

Oxidative stress is another major contributor to the development of metabolic diseases. It is closely associated with obesity, which can both cause and result from oxidative stress [6]. Excessive consumption of fats, carbohydrates, and saturated fatty acids—especially trans-fatty acids—triggers specific internal processes, such as oxidative phosphorylation, glyceraldehyde autoxidation, protein kinase C (PKC) activation, and the activation of the polyol and hexosamine pathways. These processes lead to the increased production of superoxide, a type of reactive oxygen species (ROS), which contributes to metabolic dysfunction [6,7,8].

Obesity itself can lead to oxidative stress through several mechanisms, including chronic inflammation, inadequate antioxidant defenses, hyperleptinemia, aberrant postprandial metabolism, ROS production, and elevated nitrogen oxides (NOX) activity [6,8,9]. Inflammatory cytokines like tumor necrosis factor alpha (TNF-α), interleukin-1β (IL-1β), and interleukin-6 (IL-6) are released from the adipocytes of obese individuals. These cytokines activate redox-sensitive transcription factors, such as nuclear factor-kappa B (NF-κB) and activator protein-1 (AP-1), which further increase ROS production and create a vicious cycle of inflammation and oxidative stress [7,10,11].

Obesity and metabolic syndrome are closely related, as obesity significantly increases the risk of inflammation and oxidative stress. This, in turn, raises the risk of several disorders associated with metabolic syndrome, including hypertension, insulin resistance, and hyperlipidemia [3]. Central obesity, abdominal or visceral fat accumulation, is one of the main contributors to insulin resistance and metabolic syndrome [12,13]. The low-grade inflammation associated with obesity can impair insulin signaling, causing insulin resistance, and subsequent metabolic problems [2]. Insulin resistance impairs glucose metabolism and forces pancreatic β cells to increase insulin production, leading to hyperinsulinemia and β cell hypertrophy [14,15].

Over time, insulin resistance contributes to endothelial dysfunction, visceral obesity, hyperglycemia, hypertension, dyslipidemia, and chronic inflammation, which increase the risk of developing type 2 diabetes mellitus (T2DM), metabolic syndrome (MetS), and nonalcoholic fatty liver disease (NAFLD) [3,15,16].

Adipose tissue and the liver are the primary locations of carotenoid accumulation [17,18]. Due to its lipophilic nature, lycopene is found in greater concentration in adipose tissue than in serum [19]. This tissue metabolism can be influenced by carotenoids and their metabolites [20]. Obesity has been linked to low blood concentrations of carotenoids and nutritional deficits in carotenoids [21,22].

One of the most prevalent carotenoid in the human body is lycopene [23,24]. Lycopene is not synthesized by the human body. It must be supplied by the diet [25]. Tomatoes and tomato-based products are the most common sources of this component in the human diet [26].

Most of the lycopene consumed comes from natural sources [27]. To increase its bioavailability, chewing and peristalsis are essential to break down the food, helping release lycopene from the food matrix. In the stomach, digestive enzymes, stomach acid, and mechanical movements further aid in this release [28,29]. Lycopene is then absorbed into lipid droplets and moves into the small intestine, where bile acids and enzymes continue to break it down [29].

In the small intestine, lycopene is incorporated into lipid micelles and absorbed by enterocytes, either through passive diffusion or with the help of scavenger receptor class B type 1 (SR-B1 receptors), which also absorb other carotenoids like lutein and beta-carotene [30,31,32,33]. Some of the lycopene is cleaved by β carotene oxygenase 1 (BCO_1_) and ß-carotene oxygenase 2 (BCO_2_) in the intestine [34], but most of it remains unchanged and is absorbed into chylomicrons, passing into the lymphatic system [30,35]. The enzyme microsomal triglyceride transfer protein may help deliver lipids to the chylomicrons [31]. From the lymph, lycopene enters the bloodstream, where lipoprotein lipases break down chylomicron remnants before being cleared by the liver [36].

Lycopene absorption is influenced by lifestyle and biological factors like age, gender, blood lipid levels, hormone status, body composition, diet, and smoking and alcohol consumption [37]. As a lipophilic compound, lycopene absorption is enhanced by the presence of fat but hindered by dietary fiber and beta-carotene [19,38]. After absorption, lycopene is stored mainly in the liver, prostate, and adrenal glands, but also in smaller amounts in the brain, skin, and adipose tissue [17,18,27,39]. Lycopene is metabolized in the liver, where oxidative and enzymatic processes produce active metabolites like apo-lycopenals and apo-lycopenones [40,41].

**Figure 1:**
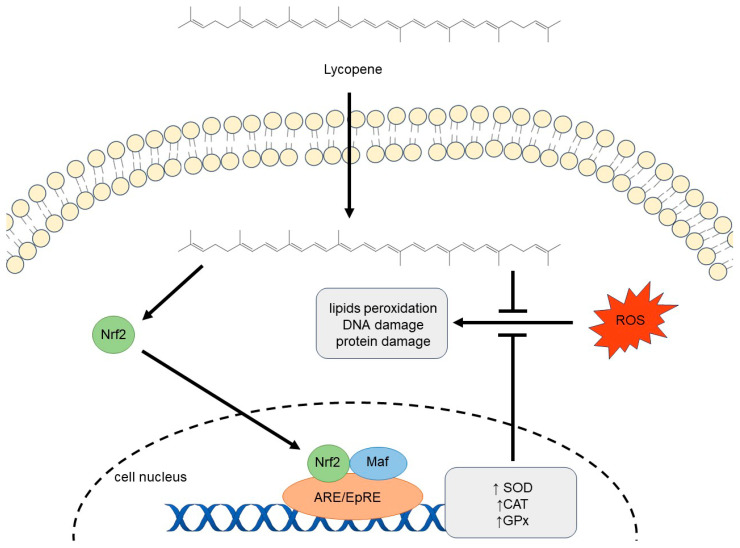
Mechanisms of lycopene′s antioxidant effect. The all-trans molecular structure of lycopene, as seen in the figure, is the most common.

Lycopene’s molecular formula is C_40_H_56_ [42]. It is a linear hydrocarbon with eleven conjugated and two non-conjugated double bonds [43]. These bonds can isomerize due to heat, light, and chemical reactions, forming 5-cis, 9-cis, 13-cis, and 15-cis [27,44,45]. The most common molecular structure is all-trans, as seen in Figure 1. Lycopene in nature has 72 cis-trans configurations [46,47]. The most stable is 5-cis lycopene, followed by all-trans, 9-cis, 13-cis, 15-cis, 7-cis, and 11-cis [47]. Isomerization impacts its activity and bioavailability, with cis and trans forms having different properties [48,49].

Lycopene from natural sources is mainly in the trans form, but in the human body, it changes to cis isomers due to food processing, storage, and metabolism [27,50]. Heat treatment increases lycopene bioavailability by converting the trans form to cis, which helps release it from the plant matrix [38,51]. Cis isomers are more soluble in bile acids and are absorbed better in the colon, and their smaller crystal size makes them more easily absorbed into the bloodstream [41,51,52].

Lycopene has antioxidant activity. It removes singlet oxygen with the greatest efficiency among all carotenoids [53]. Lycopene may quench singlet oxygen twice as effectively as beta-carotene and ten times more effectively than α-tocopherol [54]. It may remove hydrogen peroxide, nitrogen dioxide, hydroxyl radicals, singlet oxygen, and ROS [55,56]. It affects reactive oxygen species through electron transfer, radical attachment, and allylic hydrogen abstraction [55]. Free radicals and lycopene can interact in a variety of ways [55,57].

Lycopene can activate the antioxidant defense system by regulating the nuclear factor erythroid 2-related factor 2 (Nrf2) pathway. It interacts with cysteine residues on the protein Keap1, which triggers the release of Nrf2. Lycopene’s metabolites may also activate various kinases (mitogen-activated protein kinases (MAPKs), phosphatidylinositol 3-kinase (PI3K), PKC, and extracellular signal-regulated kinase (ERK)) that contribute to Nrf2 release and its nuclear translocation. In the nucleus, Nrf2 binds to small Maf proteins and attaches to antioxidant response elements (AREs), initiating the expression of antioxidant enzymes, such as glutathione peroxidase (GPx), superoxide dismutase (SOD), and catalase (CAT), increasing their levels within the cell. Through this process, lycopene indirectly enhances the body’s internal antioxidant defense by boosting the expression of these protective enzymes [58,59]. Furthermore, non-enzymatic antioxidants like vitamins C and E can be renewed by lycopene—the cellular antioxidant defense system benefits from this [53,60]. Because of its antioxidant qualities, lycopene can shield crucial bodily components such as DNA and lipids [61]. The mechanisms of lycopene′s antioxidant effect are shown in Figure 1.

Lycopene’s antioxidant properties mainly come from its chemical structure, especially the system of conjugated double bonds. The cyclic or acyclic end groups have less influence [53]. Among the various isomers of lycopene, the 5-cis form shows the strongest antioxidant effect, followed by 9-cis, 7-cis, 13-cis, 11-cis, and all-trans [62]. This is likely due to the cis forms having better solubility and less self-aggregation in polar environments [40].

Several factors affect lycopene’s reactivity within biological systems, such as its physical and molecular structure, concentration, interaction potential with other antioxidants, oxygen levels, and location within the cell [63]. In nonpolar environments, lycopene mainly forms adducts and undergoes allylic hydrogen abstraction, while electron transfer occurs in polar environments [55]. Lycopene also functions as a scavenger of superoxide radicals (O_2_^•−^) and singlet oxygen (^1^O_2_) [64].

Lycopene has an anti-inflammatory effect. Many studies in animal models and humans indicate its beneficial effects in preventing and combating inflammation in metabolic diseases [65]. It affects the regulation of signaling pathways involving the formation of inflammatory mediators [66]. It reduces pro-inflammatory cytokines: IL-6, IL-1β, and TNF-α [67,68]. It reduces the expression of the pro-inflammatory mediators cyclooxygenase 2 (COX-2) and inducible nitric oxide synthase (iNOS). It also reduces nitric oxide (NO) production [69]. Through its antioxidant activity, lycopene inhibits the expression of nuclear factor kappa B (NF-κB). Thus, it inhibits the formation of pro-inflammatory cytokines [70,71]. Lycopene may also decrease inflammation by reducing oxidative stress [72].

The biological action of lycopene is broad. Numerous studies demonstrate the protective benefits of lycopene-containing tomato-based products and lycopene against various chronic illnesses [73,74].

Chronic inflammation and oxidative stress are linked to several illnesses. Researchers are curious about the potential health benefits of lycopene as an agent that mitigates various ailments connected with metabolic diseases. This review aims to provide the findings from studies on lycopene’s impact on metabolic syndrome, obesity, and type 2 diabetes mellitus.

## 2. Methods

Electronic databases, including PubMed, Embase, and Google Scholar, were utilized to search for in vitro, in vivo, and clinical studies that explored the potential health benefits of lycopene in relation to metabolic syndrome, obesity, and type 2 diabetes. Keywords used for the literature search were “lycopene”, “metabolic syndrome”, “obesity”, “type 2 diabetes mellitus”, and their combinations. The relevance of the articles was determined by reviewing their abstracts. The manuscript includes relevant articles on the benefits of lycopene, focusing on improved metabolic health, as well as its antioxidant and anti-inflammatory effects, published between 2010 and 2024.

## 3. Metabolic Syndrome

Metabolic syndrome (MetS) is not a disease in itself. There are many definitions of MetS, but the one most frequently used to evaluate this condition is the National Cholesterol Education Program’s Adult Treatment Panel III (ATP III) report [75]. According to this definition, MetS is diagnosed by the presence of three or more of the following factors: blood glucose > 5.6 mmol/L (100 mg/dL) or drug treatment for hyperglycemia; high-density lipoprotein (HDL) < 1.0 mmol/L (40 mg/dL) in men, <1.3 mmol/L (50 mg/dL) in women or treatment for low HDL; triglycerides > 1.7 mmol/L (150 mg/dL) or treatment for hypertriglyceridemia; waist circumference > 102 cm in men or >88 cm in women; blood pressure > 130/85 mmHg or treatment for hypertension [76].

The presence of metabolic syndrome is associated with an increased risk of developing cardiovascular disease and diabetes [77]. Compared to individuals without the syndrome, those with MetS have a 2-fold increased risk of developing cardiovascular disease within the next five to ten years; a 5-fold increased risk of type 2 diabetes mellitus; a 2–4-fold increased risk of stroke; a 3–4-fold increased risk of myocardial infarction; and a 2-fold increased risk of dying [78]. Globally, metabolic syndrome is a significant public health issue [79]. According to estimates, the prevalence of MetS varies between <10% and up to 84% worldwide, depending on the area, age, sex, race, and ethnicity studied, as well as the criteria used to categorize patients. As a result, it is thought that 25% of adults worldwide suffer from MetS [78].

One of the most critical factors contributing to this syndrome is chronic inflammation occurring systemically [77]. Several current studies indicate a connection between inflammation and MetS. Slagter et al. [80] provided evidence about the adverse effects of obesity on quality of life, demonstrating that the degree of obesity, MetS, T2DM, and inflammation worsens the quality of life and that these factors are mostly linked to worse physical health [80]. Higher body mass index (BMI) was linked to lower levels of inflammation in individuals with MetS, as well as insulin resistance and higher atherogenic dyslipidemia. A positive correlation between BMI, IL-6, and pro-inflammatory C-reactive protein (CRP) supports this thesis [81]. Marques-Rocha et al. [82] demonstrated that an 8-week intervention using a Mediterranean diet altered the expression of miR-155-3p and let-7b in white blood cells in individuals with MetS. The Mediterranean diet significantly impacted the expression of microRNAs (miRNAs) linked to inflammation. The control of inflammatory genes has been linked to the production of these miRNAs, but they have also been shown to play a role in the development of human metabolic diseases [82].

Numerous studies have found that patients with metabolic syndrome had reduced plasma antioxidant enzyme activity and higher levels of oxidative stress biomarkers compared to healthy people [3].

Lifestyle changes and reduced exposure to risk factors are recommended for people at risk of developing this condition [83]. Research has confirmed that engaging in physical activity and calorie restriction contributes to reducing the risk factors for metabolic syndrome [84,85,86]. A critical factor in the development and treatment of the disease is diet. Dietary products directly impact the body’s metabolic functions, including blood pressure, cholesterol, glucose metabolism, and distribution of body fat [87]. Studies have demonstrated the potential benefits of some nutraceuticals in the management of metabolic syndrome [83]. One such ingredient is lycopene. Many studies indicate its beneficial effects in preventing and treating metabolic syndrome.

Albrahim et al. [88] conducted a study on male Wistar rats showing that lycopene helped prevent issues associated with obesity. It stopped weight growth and increased liver weight by reducing blood cholesterol, apolipoprotein B (Apo-B), triglycerides (TG), and LDL, while raising serum HDL levels. It also lowered glucose and insulin levels and improved lipid metabolism by raising hepatic peroxisome proliferator-activated receptor gamma (PPAR-γ) levels. Lycopene also prevented obesity-induced oxidative stress, inflammation, and fibrosis in the liver. This activity was caused by raised levels of antioxidant enzymes (SOD, CAT, glutathione (GSH), GPx, and glutathione reductase (GR)), lowered malondialdehyde (MDA) and NO levels, suppressed inflammatory markers (IL-1β, TNF-α, and myeloperoxidase (MPO)), and decreased fibrosis markers (alpha-smooth muscle actin (α-SMA) and transforming growth factor-beta 1 (TGF-β1)) in the liver. Additionally, by reducing blood levels of creatine kinase, lactate dehydrogenase (LDH), and the atherogenic index, lycopene reduced obesity-induced cardiac problems [88]. Fenni et al. [89] conducted a study that assessed the effect of lycopene and tomato powder supplementation on obesity-induced inflammation in C57BL/J6 mice. The consumption of the tested ingredients led to a decrease in adipocyte hypertrophy and the expression of the PPAR-γ gene, considered a key regulator of adipogenesis. This explains the reduction in obesity in mice that received lycopene and tomato powder. Furthermore, the transcription factors SREBP-1c and FAS gene levels were decreased. The researchers observed a reduction in pro-inflammatory cytokines (TNF-α, IL-6) and chemokines (CCL2 and CCL5). This might be linked to a decline in the phosphorylation of two crucial NF-κB signaling components, IkB and p65. The results suggest that the ability of lycopene and tomato powder to block NF-κB signaling in adipose tissue accounts for their anti-inflammatory effects on this tissue [89]. Ugwor et al. [90] investigated the effect of lycopene on obesity-induced cardiometabolic changes in female albino rats. A Western-style diet was used to induce obesity. Lycopene treatment decreased lipid concentrations and restored lipid and lipoprotein metabolism. The carotenoid increased nitric oxide levels and IL-10 messenger RNA transcripts and inhibited the production of mediators that promote inflammation (NF-κB-p65, IL-1β, and IL-6). Lycopene also reduces the cardiopathological harm caused by obesity [90].

There are also human studies showing the effect of lycopene on metabolic syndrome. Table 1 shows the human studies reported in this article. Yeo et al. [91] investigated whether there was an association between arterial stiffness, antioxidant levels (lycopene, β-carotene, α-tocopherol), and MetS risk. Lycopene concentrations fell as the number of risk factors for MetS increased. Serum lycopene concentrations and risk variables (waist circumference, blood pressure, triglycerides, fasting glucose, and HOMA-IR) were found to be inversely correlated. Only waist circumference, triglycerides, and HOMA-IR showed significant relationships once covariates were taken into account. The investigation results indicated a connection between MetS, brachial-ankle pulse wave velocity, and circulating lycopene. A significant rise in brachial-ankle pulse wave velocity in MetS may be linked to decreased lycopene levels [91]. Liu et al. [92] investigated the relationship between serum carotenoid concentrations (lycopene, α-carotene, β-carotene, β-cryptoxanthin, and lutein/zeaxanthin) and the prevalence of MetS in Chinese people. The prevalence of MetS in the subjects studied was estimated at 11.4%. People with MetS, compared to those without the syndrome, had increased waist circumference, BMI, blood pressure, fasting blood glucose, and triglycerides, as well as lower HDL-C concentrations. Participants without MetS had higher α-tocopherol and retinol concentrations than those with the syndrome. The study showed an inverse relationship between serum carotenoid concentrations and the incidence of MetS [92]. Han et al. [93] investigated how BMI affects the association between serum lycopene concentrations and metabolic syndrome. Participants with the lowest lycopene concentrations had a higher incidence of MetS than participants in other groups with higher lycopene concentrations. The study confirmed that BMI influenced the association between serum lycopene concentrations and the incidence of metabolic syndrome. However, this association was only significant in patients who were normal-weight and overweight. In obese subjects, this relationship was not confirmed [93]. Choi and Ainsworth [94] evaluated the relationship between MetS risk, food intake, serum vitamins and antioxidant levels, and physical activity in adults. The study showed that translycopene concentrations were positively related to the daily number of steps. The group with the lowest number of steps had the lowest serum lycopene levels, while the most active group had the highest. People with sedentary lifestyles had a higher risk of developing MetS than those who were active. The study’s findings suggest that a decreased chance of developing MetS and an increased number of steps are linked to elevated blood levels of carotenoids, especially lycopene. However, it should be noted that moderately and highly active people consumed a higher number of high-value foods than inactive people [94]. Han et al. [95] conducted a study to determine whether lycopene is related to mortality among people with MetS. People with the highest lycopene concentrations had longer mean survival times than those with the lowest serum concentrations of this carotenoid. The results of the study imply that among people with metabolic syndrome, serum lycopene concentrations are linked to a lower risk of death [95]. Bouayed et al. [96] conducted a study to investigate the association between carotenoid intake and metabolic syndrome. The study included 1346 participants. 27.1% of the individuals had metabolic syndrome. Carotenoid intake was determined by linking findings, primarily using the United States Department of Agriculture (USDA) food databases. Intake of carotenoids varied in its impact on metabolic state, risk, and syndrome, as well as the cardiometabolic components of it. Lycopene showed a somewhat favorable correlation with MetS scores and its constituent parts. Even so, these negative effects vanished for lycopene when consumption of tomato-based convenience meals was taken into account, suggesting a rather unhealthy and westernized diet [96]. Tsitsimpikou et al. [97] investigated how people with metabolic syndrome’s risk status were affected by supplementing with tomato juice. The study did not state the precise amount of tomato juice, but it did state that an average of 2.51 mg of lycopene was present in 100 g of tomato drink. There was a rise in HDL cholesterol and a decrease in LDL cholesterol. Furthermore, a noteworthy reduction in the fasting insulin resistance score was noted within the therapy group. Patients who took tomato juice supplementation showed a marked reduction in inflammation and endothelial dysfunction. The study’s findings indicate tomato juice’s potential moderating influence on risk variables linked to metabolic syndrome. The absence of a defined quantity of tomato juice and its lycopene concentration is one of the study’s limitations [97]. Li et al. [98] investigated how supplementing with tomato juice affected adipokine profiles and metabolic health-related indicators in people who were otherwise in good health. Supplementing with tomato juice significantly lowered blood levels of cholesterol, thiobarbituric reactive chemicals, monocyte chemoattractant protein-1 (MCP-1), body weight, body fat, waist circumference, and BMI. Triglycerides, lycopene, and adiponectin increased in the serum at the same time. To ensure that these findings weren’t impacted by notable drops in body weight, BMI, or body fat, a subanalysis was done. The results for the MetS variables remained significant even after splitting the subjects into responders (fat decrease) and non-responders (no fat loss). The findings indicate that in young, healthy women, taking tomato juice supplementation on a regular basis lowers waist circumference, serum cholesterol, and inflammatory adipokine levels. Body fat variations have no bearing on these outcomes. The lack of a control group in the trial was due to the authors’ observation that creating a realistic placebo drink would be impossible. Furthermore, the fact that only women of normal weight took part in the study may have hampered the credibility of the findings [98]. Mirahmadi et al. [99] conducted a randomized, double-blind, objective clinical trial to examine the effects of lycopene on oxidative stress, inflammatory markers, and liver enzymes in individuals with metabolic syndrome. Lycopene reduced CRP and prooxidant-antioxidant balance (PAB) levels. No differences were observed in alanine aminotransferase (ALT), aspartate transferase (AST), and alkaline phosphatase (ALP) levels [99].

Numerous animal and human studies provide compelling evidence of its beneficial effects in MetS, specifically targeting the core components of this condition, including dyslipidemia, inflammation, oxidative stress, insulin resistance, abdominal obesity, and elevated blood pressure.

Animal models have demonstrated that lycopene supplementation can alleviate metabolic disturbances associated with metabolic syndrome. Lycopene reduces oxidative stress and inflammation, both key drivers of insulin resistance and lipid abnormalities. Its antioxidant properties neutralize ROS, thereby protecting cellular components from oxidative damage, which is a common feature in metabolic disorders. Furthermore, lycopene has been shown to modulate lipid metabolism, enhancing lipid profiles by lowering levels of LDL and triglycerides while boosting high-density lipoprotein HDL. It also downregulates pro-inflammatory cytokines, mitigating chronic inflammation—a hallmark of metabolic syndrome. Additionally, lycopene influences gene expression related to lipid metabolism, insulin sensitivity, and inflammation. This is a beneficial action because abnormalities in the lipid profile characterize MetS.

Cross-sectional studies across various populations support these findings, showing an inverse relationship between blood lycopene levels and the risk of developing metabolic syndrome. Higher lycopene concentrations have been linked to lower insulin resistance rates, abdominal obesity, and hypertension, symptoms characteristic of MetS. Moreover, human interventional studies have confirmed that lycopene supplementation can significantly improve lipid profiles, reduce inflammatory markers such as CRP, and enhance overall metabolic function. For example, some studies report improvements in insulin sensitivity and reductions in blood pressure following lycopene supplementation.

Despite these promising results, some studies have yielded inconsistent outcomes. Variability in the effectiveness of lycopene could be attributed to differences in study designs, dosages, duration of supplementation, and the bioavailability of lycopene in different forms. Additionally, individual factors such as genetics, baseline health status, and lifestyle may influence the responsiveness to lycopene supplementation.

Lycopene holds considerable promise as a natural agent in the prevention and management of metabolic syndrome, with numerous studies demonstrating its ability to combat key features of the condition. However, methodological limitations and variations across studies necessitate further clinical research to fully elucidate its role and optimize its therapeutic potential.

## 4. Obesity

The excessive build-up of fat throughout the body or in specific organs is defined as obesity [100]. A person is considered obese if their body mass index (BMI), which is calculated by dividing their weight by the square of their height, is more than or equal to 30. Overweight people have a BMI between 25 and 29.9 [101]. It is a chronic, progressive, and recurring illness that harms both metabolic and psychological well-being [100]. There is a correlation between a number of risk factors and the elevated risk of obesity, including diets, gut microbiota, aging, genetics, and environmental variables [102]. The primary cause of obesity is an imbalance between stored and expended energy, which disrupts normal food signaling and energy use [103].

One of the main characteristics of obesity and the comorbidities that go along with it is thought to be chronic low-grade or metabolic inflammation [104,105,106]. Chronic low-grade inflammation is associated with impaired production of cytokines, acute-phase proteins, chemokines, miRNA, and other immune response mediators, as well as the induction of inflammatory signaling pathways [104,107,108,109,110,111]. An important factor in preserving this condition is adipose tissue [112].

In obese individuals, macrophages in adipose tissue contribute significantly to inflammation. Pro-inflammatory (M1) macrophages promote fat accumulation and liver inflammation, while anti-inflammatory (M2) macrophages have protective effects [113]. M1 macrophages produce pro-inflammatory factors such as IL-6, IL-12, and TNF-α [114], while M2 macrophages secrete anti-inflammatory cytokines such as vascular endothelial growth factor (VEGF), IL-10, and arginase-1 (Arg-1) [115]. Shifting macrophage polarization from M1 to M2 can reduce inflammation in adipose tissue [113,116].

Studies suggest that lycopene may aid in the treatment of obesity due to its anti-inflammatory effects on adipose tissue [117,118,119]. Many studies in animal models and humans indicate its beneficial effects in preventing and fighting obesity.

Lycopene showed antioxidant and anti-inflammatory activity in male Sprague-Dawley rats with nonalcoholic steatohepatitis fed a high-fat diet. The tested carotenoid reduced the level of TNF-α in serum. It also lowered MDA levels, as its supplementation significantly inhibited lipid peroxidation induced by a high-fat diet, restoring serum and liver MDA levels to values similar to those of the control group. Lycopene also increased the concentration of GSH in the liver [120]. It also improved tissue insulin resistance. Alanine aminotransferase (ALT) and triglyceride levels decreased. Hepatic steatosis and inflammation in the liver decreased under the influence of the tested carotenoid. A reduction in serum TNF-α levels was also observed. The expression of α-smooth muscle actin (α-SMA) and cytochrome P450 2E1 (CYP 2E1) decreased in rats fed lycopene. There was no significant difference among the different doses of lycopene and the effects obtained [120]. In C57BL/6J mice fed a high-fat diet, lycopene attenuated glucose intolerance and hyperinsulinemia. The researchers also observed reduced adipocyte hyperplasia and macrophage infiltration in the epididymal white adipose tissue, as well as inflammation and hepatic steatosis. The carotenoid reduced the number of adipose tissue macrophages and also had an effect on M1 and M2 polarization—M2 predominance over M1 was observed in the studied macrophages. In adipose tissue macrophages, lycopene promotes dominant M2 polarization. This process reduces inflammation and insulin resistance in the liver and epididymal white adipose tissue caused by a high-fat diet [121]. In a 12-week trial, lycopene decreased obesity and weight gain in Swiss white mice given a high-fat diet. It reduced systemic obesity and total serum triglycerides, improved hepatic glucose and lipid metabolism, and accelerated glucose clearance and insulin sensitivity. All of these actions contributed to improving adipose tissue mobilization and reducing insulin resistance [122]. Kim et al. [123] showed that lycopene added to tomato wine inhibited body weight growth in male Sprague-Dawley rats fed a high-fat diet without lowering food consumption. Another study in C57BL/6J mice showed that lycopene supplementation prevented the development of obesity. Lycopene reduced fat storage in adipose tissue and supported lipid metabolism by blocking the expression of genes responsible for lipogenesis (Acaca, Fas, Pparγ, Srebp1c, and Pparg) and activating genes related to lipidolysis and thermogenesis (Pgc1α, Prdm16, Ucps, and Ebf2), as well as mitochondrial function (Sirt1, Cox5b, Cox8b, CoxII, and Cycs). Lycopene also inhibited autophagy-dependent lipid accumulation by reducing the expression of autophagy-related genes (Atg7, Atg14, Lc3, P62, and Beclin). Additionally, it improved insulin sensitivity by reducing leptin levels and increasing the expression of glucose transporters, Glut1 and Glut4. Lycopene also reduced inflammation and intestinal leakiness by reducing the expression of inflammatory biomarkers (IL-1β, IL-6, iNOS, TNF-α, and Cox-2) and enhancing the expression of proteins responsible for intestinal barrier integrity, such as Zo-1, claudin-1, and occludin [124]. Zeng et al. [125] found that lycopene significantly prevented insulin resistance in mice fed a high-fat diet. It lowered blood glucose and insulin levels, improved glucose and insulin tolerance, and increased liver glycogen content. Lycopene also reduced inflammation by inhibiting the increase in IL-1β, TNF-α, and CRP levels. It improved lipid profiles by reducing total cholesterol, triglycerides, and LDL levels and increasing HDL levels. Additionally, lycopene inhibited STAT3 expression and phosphorylation in the liver and blocked STAT3 signaling and Srebp-1c gene expression, which prevented inflammation, lipid accumulation, and metabolic dysfunction [125]. Lorenz et al. [126] found that lycopene administered to male New Zealand White rabbits fed a high-cholesterol diet reduced serum total cholesterol and LDL cholesterol levels and also reduced aortic cholesterol ester levels [126]. Researchers administered lycopene to male Wistar rats on a hyperenergetic diet to examine its effects on adipokine expression in obesity. Supplementing with lycopene significantly lowered plasma levels of resistin, leptin, and IL-6 gene expression in epididymal adipose tissue, but it had no effect on body weight or adiposity. The expression of the MCP-1 gene in epididymal adipose tissue was likewise markedly decreased. According to the study, lycopene might be a useful tactic for lowering inflammation in obesity [127]. Luvizotto et al. [128], in a study on male Wistar rats fed a hypercaloric diet, observed that lycopene, through its effect on adipose tissue, may play a role in preventing complications associated with obesity. After the administration of the carotenoid to rats, they observed an increase in its concentration in plasma and in the expression of adiponectin mRNA in adipose tissue. In fat cells of obese rats, lycopene treatment also enhanced the expression of SIRT1, FoxO1, and FAT/CD36 mRNA but lowered the expression of PPARγ [128].

However, some researchers found that administering lycopene to obese animals did not impact body weight or the adiposity index [127,128,129,130]. This inconsistency might be due to animal models, a form of lycopene and its carrier, variations in dosage, and treatment duration.

There are also human studies on the effects of lycopene on obesity. These trials are summarized in Table 2. In a cross-sectional study, Harari et al. [131] discovered a negative correlation between blood levels of CRP, waist circumference, body fat, and BMI, and the concentration of lycopene in the blood. Han et al. [132] found that higher blood uric acid levels were associated with hypertension in adults with BMI ≥ 25. Blood lycopene levels were inversely associated with hypertension. Additionally, the lycopene to uric acid ratio was significantly associated with hypertension in overweight and obese individuals [132]. McMorrow et al. [133] discovered that a dietary supplement including, among others, lycopene preserved high molecular weight adiponectin levels and decreased insulin resistance as measured by homeostatic model analysis (HOMA-IR) in a randomized, controlled crossover trial of overweight teenagers. This resulted from the methylation of adipogenic genes being modulated in both directions [133]. Negri et al. [134] conducted a randomized crossover clinical trial to examine the effect of lycopene-rich tomato juice in obese children. Tomato juice supplementation enhanced lipid and glucose metabolism. This was linked to the reduction of inflammation and oxidative stress, as well as to its impact on T lymphocyte mitochondrial metabolic control [134]. Park et al. [135] showed in their study that serum lycopene levels are negatively correlated with BMI in overweight children aged 9–10. Ghavipour et al. [136] investigated the effect of tomato juice consumption on markers of oxidative stress in overweight and obese women. In overweight individuals, after 20 days of tomato juice intake, the researchers observed significant increases in plasma total antioxidant capacity (TAC), erythrocyte SOD, GPx, and CAT. They also observed decreases in serum MDA. However, in the obese group, these changes were not statistically significant [136].

Wiese et al. [137], in a randomized, double-blind, 1-month study, examined the effects of lycopene on middle-aged adults with moderate obesity (BMI between 30 and 35). Supplementing with lycopene enhanced the relative number of lactobacilli, bifidobacteria, and other beneficial gut microorganisms. Improvements in skeletal muscle oxygenation and hepatic lipid metabolism were noted by the researchers. Additionally, they observed a decrease in skin corneocyte desquamation and an increase in skin sebum viscosity. This study demonstrated the prebiotic potential of lycopene [137].

On the other hand, Ben Amara et al. [138] conducted a cross-sectional observational research with 108 obese, non-diabetic participants and found no correlation between plasma lycopene levels and BMI, adipokines, or insulin resistance. Their inability to account for dietary composition using a validated eating pattern questionnaire, however, could have compromised their capacity to estimate carotenoid consumption precisely. Furthermore, the study’s limited sample size, imprecise definition of obesity, and absence of an age- and sex-matched control group could have compromised lycopene’s reported therapeutic advantages [138].

Lycopene exhibits significant potential in combating obesity-related diseases through its antioxidant, anti-inflammatory, and metabolic-regulating properties. Several studies have explored the effects of lycopene in animal models and human trials, demonstrating its beneficial impact on obesity and related metabolic dysfunctions.

In animal studies, lycopene supplementation reduced inflammation, improved insulin sensitivity, and reduced fatty liver disease in rodents fed a high-fat diet. Lycopene’s effects extend beyond simple inflammation reduction. It regulates gene expression involved in lipid metabolism, blocking genes responsible for fat accumulation (Acaca, Fas, Pparγ) and enhancing lipid breakdown and thermogenesis via upregulation of genes such as Pgc1α, Prdm16, and Ucps. It also inhibits autophagy-related lipid accumulation, reducing the expression of genes like Atg7, Atg14, and Lc3. Additionally, lycopene improves insulin sensitivity by increasing the expression of glucose transporters (Glut1, Glut4) and reducing leptin levels, highlighting its role in improving both lipid and glucose metabolism in obesity models.

Human studies corroborate many of these findings. Cross-sectional studies have shown inverse correlations between blood lycopene levels and markers of obesity, such as waist circumference, BMI, and CRP. Lycopene supplementation has been associated with improved lipid profiles, reduced inflammation, and enhanced glucose metabolism in various clinical trials.

Lycopene’s effects are largely attributed to its potent antioxidant activity, which reduces ROS and inhibits lipid peroxidation. This antioxidant activity helps prevent oxidative damage in adipose tissue, the liver, and skeletal muscle. Furthermore, lycopene modulates inflammatory pathways by downregulating pro-inflammatory cytokines such as TNF-α, IL-1β, and IL-6, and by enhancing the expression of proteins that support intestinal barrier integrity (Zo-1, claudin-1, occludin). By improving gut health and reducing intestinal permeability, lycopene also helps prevent systemic inflammation, a key driver of obesity-related metabolic disorders.

Despite these promising results, not all studies have shown uniform effects. Some trials in both animal and human models failed to demonstrate significant changes in body weight or adiposity with lycopene supplementation. These inconsistencies could be due to variations in the animal models used, the form of lycopene administered, the dosage, and the duration of treatment. In human studies, limitations such as small sample sizes and the failure to account for dietary habits may have affected results.

In summary, lycopene’s multifaceted role in combating obesity-related metabolic disorders is supported by its ability to reduce oxidative stress, inflammation, and lipid accumulation while improving glucose metabolism and insulin sensitivity. These mechanisms highlight its potential as a therapeutic agent in managing obesity and preventing related complications. However, further research is needed to clarify optimal dosing, administration forms, and long-term effects, particularly in human populations.

## 5. Type 2 Diabetes Mellitus

Diabetes is a chronic illness that ranks among the world’s most serious public health issues [139,140]. Type 2 diabetes results from insulin resistance, decreased insulin production, or a combination of both. Over time, it can cause complications such as neuropathy, nephropathy, retinopathy, and cardiovascular disorders [141]. A key factor in insulin resistance is obesity-induced chronic inflammation [102]. This low-grade inflammation, especially in white adipose tissue, interferes with insulin signaling by activating serine and threonine kinases such as JNK, IKK, and PKC-θ. These kinases are also triggered by microbial products like lipopolysaccharides (LPS) and peptidoglycan via Toll-like receptor (TLR) pathways in obesity [78].

Oxidative stress is closely linked to the inflammatory state in type 2 diabetes [142]. It is worsened by the increased production of advanced glycation end products, which are driven by glyco-oxidation processes [143,144]. Studies show elevated levels of inflammatory markers such as TNF-α, IL-6, and CRP in individuals with type 2 diabetes [145,146]. Hypoglycemia, a hallmark of diabetes, contributes to oxidative stress through mechanisms such as glucose autoxidation and the activation of polyol and hexosamine pathways. This leads to the overproduction of ROS in the mitochondria, which damages cellular components and drives diabetes-related complications [147,148].

Numerous studies indicate a link between oxidative stress and type 2 diabetes. This connection was identified by analyzing oxidative stress biomarkers in individuals with type 2 diabetes [145,146,149,150,151,152]. Individuals with type 2 diabetes showed reduced glutathione peroxidase activity compared to the control group [146,149,150,152]. Jiffri et al. [146] and Mandal et al. [151] also reported decreased SOD activity, though Aouacheri et al. [149] noted an increase in this parameter, while Picu et al. [152] found no change in SOD activity. Additionally, patients with type 2 diabetes had lower levels of GSH and CAT [146,149,150,151,152], and levels of MDA, a marker of oxidative stress, were elevated in these patients [145,146,149,150,151]. Mandal et al. [151] also detected a reduced TAC. Hypoglycemia and oxidative stress can decrease the expression of CAT, SOD, and GSH-Px in pancreatic β cells, and prolonged oxidative stress can inhibit insulin secretion by these cells [153].

Using pharmacologically active drugs or changing one’s lifestyle might help prevent or postpone the onset of type 2 diabetes [140]. The onset and management of type 2 diabetes are significantly influenced by diet. It is important to prevent this illness. Certain dietary elements are beneficial to health [154]. Studies suggest that tomato products and lycopene may be useful in preventing and treating type 2 diabetes [155].

Imran et al. [27] described the antidiabetic activity of lycopene. This activity was connected with lowering blood levels of MDA, serum nitrate-nitrite, glycated hemoglobin, and CRP, downregulating the expression of the RAGE receptor, NF-κB, MMP-2, and Bax proteins, increasing and enhancing the activities of antioxidant enzymes, and improving Bcl-xL and Bcl-2 levels [27].

Guo et al. [156] studied the effect of lycopene on streptozotocin-induced diabetic nephropathy in male Kunming mice. Lycopene alleviated the symptoms of nephropathy. It reduced proteinuria. Researchers observed reduced levels of fasting plasma glucose, LDL in the blood, and a decrease in urinary protein. They also noted an increase in body weight and elevated levels of HDL. In addition, lycopene showed antioxidant activity. The activities of the antioxidant enzymes GPx and SOD were increased. The MDA content was reduced. An immunohistochemical study showed that lycopene had anti-inflammatory effects. It was manifested as a reduction in the expression of TNF-α and NF-κB in kidney tissue. The researchers also observed increased levels of HO-1 in the kidneys. The study found that lycopene exhibits anti-inflammatory and antioxidant effects and may show protective effects in diabetic nephropathy [156]. Li et al. [157] investigated how lycopene affected renal function in diabetic nephropathy in male Sprague-Dawley rats with streptozotocin-induced diabetes. Reduced creatinine, blood urea nitrogen, and 24-h urea protein were the results of the carotenoid. HDL rose, whereas blood lipids such as TC, TG, and LDL decreased. In diabetic renal tissues, lycopene improved SOD activity, Akt/PKB phosphorylation, and reduced MDA content and CTGF expression. By enhancing oxidative state, controlling phosphorylated Akt (p-Akt) and connective tissue growth factor (CTGF), and regulating oxidative status, lycopene prevents the development of diabetic nephropathy and improves renal function [157].

Akinnuga et al. [158] investigated the hypoglycemic effect of tomatoes containing lycopene in albino Wistar rats with streptozotocin-induced diabetes. Researchers observed a reduction in blood glucose levels after just 3 days of eating tomatoes [158]. Zhu et al. [159] investigated the effect of lycopene on oxidative stress and endothelial dysfunction in streptozotocin-induced diabetic male Wistar rats. The tested carotenoid improved endothelial function. It reduced serum glucose levels and ox-LDL levels. An increase in SOD, constitutive nitric oxide synthase (cNOS), and NO activity was also observed in the aorta. Inducible nitric oxide synthase (iNOS) activity and MDA levels in the aorta decreased. Lycopene alleviated endothelial dysfunction through its antioxidant activity [159]. In another study, scientists examined the effect of lycopene in alleviating diabetes complications in terms of its effect on angiotensin-converting enzyme activity in male Wistar-Albino male rats with streptozotocin-induced diabetes. The carotenoid caused a reduction in blood glucose and glycated hemoglobin (HbA1c) levels. It was discovered that the use of lycopene effectively inhibited angiotensin-converting enzyme (ACE) activity, a crucial marker of problems associated with diabetes [160]. In another study conducted on male Sprague-Dawley rats with streptozotocin-induced diabetes, lycopene showed antioxidant and antidiabetic effects. It lowered blood glucose levels. It reduced oxidative stress by reducing MDA levels and increasing SOD activity. The tested carotenoid also caused up-regulation of endothelial nitric oxide synthase (eNOS) expression [161]. Bayramoglu et al. [162] examined how lycopene affected the Sprague-Dawley rats’ streptozotocin-induced diabetic symptoms. The tested carotenoid had a hypoglycemic effect. It lowered glucose levels and increased serum insulin levels. It also reduced serum total cholesterol, triglycerides, aspartate aminotransferase (AST), and alanine aminotransferase (ALT) concentrations [162]. Saad et al. [163] investigated the protective effect of lycopene on pancreatic cells in male Balb/c mice with streptozotocin-induced diabetes. The carotenoid decreased ROS levels in serum, pancreas, and liver tissues. It also decreased CAT, SOD, and GPx levels. Lycopene prevented the increase in lipid peroxidation levels in the pancreas and liver [163]. Ozmen et al. [147] studied the effect of lycopene on streptozotocin-induced diabetes in Sprague-Dawley rats. The carotenoid studied did not have a negative effect on the pancreas of the test animals. It decreased vacuolization in the pancreas. The researchers observed that lycopene decreased blood and urine glucose levels. It also increased insulin levels in serum [147]. Another study evaluated the effects of lycopene on male Wistar rats with streptozotocin-induced diabetes. The researchers observed that lycopene exhibited antidiabetic properties. It decreased blood glucose levels. The carotenoid studied also showed antioxidant activity. It increased CAT, SOD, GST, and GPx levels in the liver and decreased MDA levels in the liver. The researchers observed no significant changes in the levels of red blood cells (RBC), hemoglobin, hematocrit, mean corpuscular volume (MCV), mean corpuscular hemoglobin concentration (MCHC), and mean corpuscular hemoglobin (MCH). The studied carotenoid showed positive effects on the liver. It mitigated histopathological changes in this organ. It also caused an increase in AST, ALT, ALP, and LDH activities. However, no significant effect was observed on triglycerides and total cholesterol [164]. Sharma et al. [165] studied the antidiabetic activity of lycopene niosomes in male and female Wistar rats with alloxan monohydrate-induced diabetes. The researchers observed decreased blood glucose levels. Effects on lipid profiles were also noted. Total cholesterol, triglycerides, LDL, and VLDL levels were reduced [165]. In another study, researchers analyzed the effects of lycopene on changes in erythrocyte osmotic fragility and lipid peroxidation caused by streptozotocin-induced diabetes in male and female Wistar rats. The tested carotenoid significantly reduced erythrocyte osmotic fragility. It reduced the level of malondialdehyde in erythrocytes, which is an indicator of lipid peroxidation. Both observed effects were related to the antioxidant activity of lycopene [166]. Assis et al. [167] studied the effects of lycopene on streptozotocin-induced diabetic male Wistar rats. The researchers observed reduced glycemia. They also noted a reduction in serum ox-LDL and liver thiobarbituric acid reactive substances. The carotenoid increased SOD and CAT activity, as well as non-protein sulfhydryl group levels. However, no significant differences were observed in GPx levels. Lycopene lowered total cholesterol and triacylglycerol levels and increased HDL levels [167]. In another study, researchers administered lycopene to female Wistar rats with streptozotocin-induced diabetes. The tested carotenoid had an antioxidant effect. It increased the activity of SOD, CAT, GST, and GPx, and also decreased the level of MDA [168]. Malekiyan et al. [169] investigated the neuroprotective and antioxidant activity of lycopene in male Wistar rats with streptozotocin-induced diabetes. The tested carotenoid prevented apoptosis of hippocampal neurons. It also improved cognitive functions. Both results were related to its antioxidant activity and reduction of oxidative stress. It increased the TAC and reduced the level of MDA [169]. Yin et al. [154] investigated how lycopene affects glycolipid metabolism in male Sprague Dawley rats with streptozotocin-induced diabetes. The carotenoid reduced fasting blood glucose and the level of glycosylated hemoglobin. It increased insulin levels. It also caused a reduction in lipid levels in the liver and blood. Researchers observed increased SOD and GPx activity in the pancreas and decreased MDA levels in this organ. Lycopene had a positive effect on glycolipid metabolism and also had antidiabetic and antioxidant effects [154]. Zheng et al. [70] investigated the anti-inflammatory and antioxidant effects in male Sprague-Dawley rats with streptozotocin-induced diabetes. The tested carotenoid caused a decrease in fasting blood glucose and an increase in fasting blood insulin. It also caused a reduction in MDA, GHb, and ox-LDL levels. Researchers observed an increase in SOD, CAT, and GPx activity. There was also a noticeable reduction in inflammatory factors: CRP and TNF-α. In the study, lycopene showed antioxidant, anti-inflammatory, and antidiabetic effects [70].

There are also human studies on the effects of lycopene on type 2 diabetes. Table 3 summarizes those trials. A study that used 24-h meal recalls and health checks on adult Koreans showed that non-diabetic patients consumed more lycopene than participants with diabetes [170]. However, another prospective cohort study conducted in the Netherlands found no correlation between a diet containing lycopene and the occurrence of type 2 diabetes mellitus [171]. Li et al. [172] examined serum lycopene levels in patients with type 2 diabetes mellitus who have diabetic retinopathy and those without the condition. Lycopene levels were lower in people with diabetes than in patients without the disease. Patients with diabetes and with proliferative diabetic retinopathy had lower levels of this carotenoid than those without diabetic retinopathy or with non-proliferative diabetic retinopathy. Hemoglobin A1c was negatively associated with serum lycopene concentration [172]. She et al. [173] examined the association between serum levels of carotenoids, including lycopene, and the risk of diabetes and diabetic retinopathy. However, the study found that there was no significant association between glycated hemoglobin (HbA1c) and lycopene. Serum lycopene levels were comparable in the study groups, both in healthy individuals and those with diabetes [173]. Leh et al. [62] investigated how lycopene consumption affected the glycemic state and antioxidant capability of individuals with type 2 diabetes mellitus. They observed that the intake of the studied carotenoid was positively associated with peripheral antioxidant levels. An inverse relationship was observed for HbA1c and fasting plasma glucose (FPG). Their levels decreased with lycopene consumption [62].

Through the regulation of many signaling pathways, as well as its anti-inflammatory and antioxidant properties, lycopene lowers the chance of developing type 2 diabetes and has a positive impact on its treatment [117,174].

Studies reveal that lycopene lowers markers of oxidative stress such as MDA, serum nitrate-nitrite, and HbA1c, while downregulating pro-inflammatory proteins such as NF-κB, RAGE receptor, and Bax, and increasing the expression of anti-apoptotic proteins like Bcl-2 and Bcl-xL [156,157,158,159,165,167]. Additionally, lycopene enhances antioxidant enzyme activity, such as SOD and GPx, thereby improving overall oxidative status in tissues affected by diabetes [156,157,161,167,173].

In animal diabetes models, lycopene exerted a hypoglycemic effect, reducing blood glucose and HbA1c levels. These effects are largely attributed to its ability to regulate oxidative stress and inflammatory pathways, such as by reducing angiotensin-converting enzyme activity and increasing endothelial nitric oxide synthase expression [160,161,166,169].

Lycopene’s anti-inflammatory and antioxidant properties are central to its antidiabetic action. For instance, it reduces ROS in pancreatic and liver tissues, inhibits lipid peroxidation, and upregulates crucial antioxidant enzymes like SOD and CAT, protecting organs such as the pancreas, liver, and kidneys from oxidative damage [156,163,164,167].

Animal studies provide substantial evidence of lycopene’s protective role in diabetic complications. It showed a positive effect in treating diabetic nephropathy. This effect was observed through the reduction of proteinuria, creatinine, and urea nitrogen in serum. In diabetic nephropathy, its regulation of CTGF and Akt phosphorylation further emphasizes its role in preventing tissue damage [157,159,163,168]. Lycopene also mitigates diabetes-related neurodegeneration by preventing apoptosis in hippocampal neurons and enhancing cognitive functions [169].

Human studies offer mixed results, though several indicate a positive association between lycopene levels and reduced risk or severity of type 2 diabetes. Lower serum lycopene concentrations have been found in individuals with diabetic retinopathy and higher HbA1c levels, suggesting a protective role for this carotenoid in mitigating diabetic complications [170,172,173]. While some studies did not find a direct correlation between lycopene consumption and diabetes prevalence [171], others reported improvements in glycemic control, lipid profiles, and antioxidant capacity with lycopene supplementation [62,172].

Lycopene’s effects are mediated through several mechanisms, including the modulation of oxidative stress, inflammation, lipid metabolism, and the regulation of key enzymes and signaling pathways, making it a promising compound for the prevention and management of type 2 diabetes [154,156,174]. However, further clinical studies are needed to clearly define the role of lycopene in the treatment and prevention of diabetes and its complications.

## 6. Conclusions

Metabolic syndrome, diabetes, and obesity are closely related. Their occurrence increases the risk of other health complications, including cardiovascular diseases. Obesity leads to insulin resistance, which is a key factor in the development of metabolic syndrome. Insulin resistance promotes the occurrence of metabolic dysfunctions, including elevated blood glucose levels, which can lead to the development of type 2 diabetes. On the other hand, diabetes deepens metabolic disorders and contributes to further weight gain and increased inflammation in the body. These diseases are mutually reinforcing, which means that preventing and treating one of them can have a beneficial effect on the others. Therefore, a comprehensive approach to this problem is important. Research indicates that diet and the ingredients consumed have a major impact on the prevention and treatment of these diseases. Numerous studies in animal models and human trials suggest that lycopene may have beneficial effects on metabolic disorders. The conducted research provided evidence that lycopene may be a potential agent used in the prevention and treatment of metabolic syndrome, obesity, and type 2 diabetes.

In the case of metabolic syndrome, animal studies have shown that lycopene alleviates symptoms such as dyslipidemia, inflammation, oxidative stress, and metabolic disorders. In human studies, lycopene administration led to improved lipid profiles, reduced inflammation, and supported metabolic functions. However, not all studies were consistent with all parameters, which may be due to methodological limitations. Therefore, further clinical trials are needed to confirm its efficacy in preventing and treating metabolic syndrome.

In the case of obesity, which often coexists with metabolic syndrome and diabetes, lycopene has shown potential to reduce inflammation, improve insulin sensitivity, and reduce fatty liver disease in animal studies. In human studies, higher blood levels of lycopene have been associated with lower BMI, waist circumference, and CRP levels, which may reduce the risk of related diseases.

Lycopene also shows promising antidiabetic properties, mainly due to its antioxidant and anti-inflammatory properties. In animal studies, lycopene lowered glucose, LDL, total cholesterol, and triglyceride levels, and increased HDL levels. Although human studies are less conclusive, lycopene may have potentially beneficial effects on diabetes. However, further clinical trials are required to confirm its effectiveness and directions of action.

In summary, lycopene has promising potential in the prevention and treatment of metabolic diseases. However, further clinical trials are needed to confirm its efficacy in the successful treatment of human diseases.

## Figures and Tables

**Table 1:** Human studies evaluating lycopene’s impact on metabolic syndrome.

Participants	Study Type	MetS Definition	Lycopene Dosage	Main Results	Year	Reference
299 Korean men, 48–50 years	Cross-sectional study	Modified ATP III guidelines, American Diabetes Association guidelines, and Asian-Pacific guidelines	Dietary intake	1. Lycopene levels decreased with increasing MetS risk factors.	2011	[91]
				2. Higher brachial-ankle pulse wave velocity in MetS may be associated with lower lycopene levels.		
2148 participants from China (1547 women and 601 men), 50–75 years	Cross-sectional study	2005 International Diabetes Federation MetS diagnostic criteria	Dietary intake	1. People with MetS had higher waist circumference, BMI, blood pressure, fasting blood glucose, triglycerides, and lower HDL.	2014	[92]
				2. People without MetS had higher α-tocopherol and retinol levels.		
				3. Serum carotenoid concentrations were inversely related to MetS incidence.		
13,196 participants (6335 men and 6861 women), 20 years or older	Cross-sectional study	ATP III criteria	Dietary intake	1. Participants with the lowest lycopene concentrations had a higher incidence of MetS than those with higher lycopene levels.	2016	[93]
				2. BMI influenced the link between serum lycopene levels and MetS incidence (significant in overweight and normal-weight people, not in obese people).		
1930 participants (948 men and 982 women), 40–70 years	Cross-sectional study	ATP III criteria	Dietary intake	1. Translycopene concentrations were positively related to the daily number of steps.	2016	[94]
				2. People with sedentary lifestyles had a higher risk of developing MetS.		
				3. Elevated blood levels of carotenoids, especially lycopene, are probably linked to a lower risk of MetS and an increased number of steps.		
2499 participants with metabolic syndrome (1209 men and 1290 women), 20 years or older	Cross-sectional study	ATP III criteria	Dietary intake	1. People with metabolic syndrome and the highest lycopene levels had longer average survival times than those with the lowest levels.	2016	[95]
1346 participants (630 men and 716 women), 25–79 years	Cross-sectional study	ATP III criteria	Dietary intake	1. Carotenoid intake varied in its impact on metabolic state, risk, and MetS components.	2024	[96]
				2. Lycopene showed favorable correlation with MetS scores.		
Tomato juice group: 13 men and 2 women, control group: 11 men and 1 woman, participants with metabolic syndrome, 43–67 years	Comparative study	American Heart Association/National Heart, Lung and Blood Institute (AHA/NHLBI) definition	Once a day unspecified amount of tomato juice containing 2.51 mg of lycopene in 100 mL, four times a week, or a placebo for 2 months	1. Increased HDL.	2014	[97]
				2. Decreased LDL.		
				3. Reduced fasting insulin resistance score.		
				4. Reduced inflammation.		
				5. Reduced endothelial dysfunction.		
25 women, BMI ≥ 20, 20–30 years, no control group	Cross-sectional, single-center study	Not reported	280 mL tomato juice daily, containing 32.5 mg of lycopene for 2 months	1. Decreased cholesterol.	2015	[98]
				2. Decreased thiobarbituric reactive chemicals.		
				3. Decreased MCP-1.		
				4. Decreased body weight, body fat, waist circumference, and BMI,		
				5. Increased triglycerides.		
				6. Increased lycopene levels.		
				7. Increased adiponectin.		
Lycopene group: 18 men and 22 women, control group: 12 men and 28 women, participants with metabolic syndrome, 18–60 years	Randomized, double-blind, objective-based clinical trial	International Diabetes Federation criteria	20 mg of lycopene per day or a placebo for 8 weeks	1. Decreased CRP.	2023	[99]
				2. Decreased PAB.		
				3. No differences in ALT, AST, and ALP levels.		

ALP: alkaline phosphatase; ALT: alanine aminotransferase; AST: aspartate transferase; ATP III: National Cholesterol Education Program’s Adult Treatment Panel III; BMI: body mass index; CRP: C-reactive protein; HDL: high-density lipoprotein; LDL: low-density lipoprotein; MCP-1: monocyte chemoattractant protein-1; MetS: metabolic syndrome; PAB: prooxidant-antioxidant balance.

**Table 2:** Human studies evaluating lycopene’s impact on obesity.

Participants	Study Type	Lycopene Dosage	Main Results	Year	Reference
80 participants (31 men and 49 women), BMI: 30.1–48.5, 18–70 years	Small cohort, cross-sectional analysis	Dietary intake	1. Lycopene concentration is lower in people with obesity.	2020	[131]
8556 participants (4266 men and 4296 women), BMI ≥ 25, 20 years and older	Cross-sectional study	Dietary intake	1. Blood lycopene levels are inversely associated with hypertension.	2017	[132]
			2. The lycopene to uric acid ratio is significantly associated with hypertension in overweight and obese individuals.		
70 overweight adolescents, BMI > 31, 13–18 years	Randomized, double-blind, placebo-controlled, crossover trial	Dietary supplement including, among others, lycopene, for 8 weeks	1. Preserved high molecular weight adiponectin levels.	2018	[133]
			2. Decreased insulin resistance.		
52 obese children with fatty liver, BMI > 85th percentile, 4–14 years	Randomized, crossover, double-blind trial	100 mL of lycopene-enriched (0.011%) tomato juice daily for 60 days	1. Enhanced lipid and glucose metabolism.	2020	[134]
			2. Reduced inflammation and oxidative stress.		
			3. Impact on T lymphocyte mitochondrial metabolic control.		
34 overweight children (18 boys and 16 girls), BMI > 21, 9–10 years	Observational study	Dietary intake	1. Serum lycopene levels are negatively correlated with BMI.	2010	[135]
64 women, BMI ≥ 25, 20–30 years	Randomized controlled clinical trial	330 mL of tomato juice daily, containing 37.0 mg of lycopene or water (placebo) for 20 days	1. In overweight people: increased TAC, SOD, GPx, and CAT; decreased MDA.	2015	[136]
			2. In obese people: these changes were not statistically significant.		
30 participants (15 men and 15 women), BMI: 30–35, 40–68 years	Randomized, double-blind trial	7 or 30 mg of lycopene daily in different formulations or placebo for 1 month	1. Enhanced the relative number of beneficial gut microorganisms.	2019	[137]
			2. Improved skeletal muscle oxygenation and hepatic lipid metabolism.		
			3. Decreased skin corneocyte desquamation.		
			4. Increased skin sebum viscosity.		
108 obese, non-diabetic participants (26 men and 82 women), BMI ≥ 30, 18–70 years	Cross-sectional study	Dietary intake	1. No correlation between plasma lycopene levels and BMI, adipokines, or insulin resistance.	2015	[138]

CAT: catalase; BMI: body mass index; GPx: glutathione peroxidase; MDA: malondialdehyde; SOD: superoxide dismutase; TAC: total antioxidant capacity.

**Table 3:** Human studies evaluating lycopene’s impact on diabetes mellitus.

Participants	Study Type	Lycopene Dosage	Main Results	Year	Reference
37,846 participants (men and women) from Utrecht and its surroundings, Amsterdam, Doetinchem, and Maastricht, Netherlands	Prospective cohort study	Dietary intake	1. No correlation between a diet containing lycopene and the occurrence of type 2 diabetes.	2014	[171]
24,377 Korean adults (9779 men and 14,598 women), including 603 people with T2DM (332 men and 271 women)	Cross-sectional surveillance	Dietary intake	1. Non-diabetic patients consumed more lycopene than diabetic patients.	2017	[170]
T2DM group: 71 patients; control group: 23 patients	Case-control study	Dietary intake	1. HbA1c negatively associated with serum lycopene concentration.	2010	[172]
			2. Lower lycopene levels in people with diabetes than in those without.		
			3. Lower lycopene levels in diabetic patients with proliferative diabetic retinopathy than in those without retinopathy.		
			4. Lower lycopene levels in proliferative diabetic retinopathy compared to those in non-proliferative.		
Diabetic retinopathy group: 272 patients; diabetes mellitus without retinopathy group: 190 patients; control group: 285 patients	Cross-sectional study	Dietary intake	1. No significant relationship between HbA1c levels and lycopene.	2017	[173]
T2DM group: 87 patients; control group: 122 patients	Case-control study	Dietary intake, 0.04 mg/kg/day for 12 months	1. Fasting plasma glucose and HbA1c levels significantly decreased with increased lycopene consumption.	2021	[62]

HbA1c: glycated hemoglobin; T2DM: type II diabetes mellitus.
